# Predictive Value of MELD Score and Charlson Comorbidity Index in Thoracic Aortic Surgery Patients

**DOI:** 10.3390/jcdd12120463

**Published:** 2025-11-28

**Authors:** Ismail Dalyanoglu, Freya Sophie Jenkins, Luis Jaime Vallejo Castano, Esma Yilmaz, Mohammed Morjan, Amin Thwairan, Johanna Wedy, Georg Ulrich Holley, Artur Lichtenberg, Hannan Dalyanoglu

**Affiliations:** 1 Sankt Marien Hospital Gelsenkirchen-Buer, 45894 Gelsenkirchen, Germany; i.dalyanoglu@st-augustinus.eu; 2 Department of Cardiac Surgery, Medical Faculty, Heinrich Heine University, 40225 Dusseldorf, Germany; freyasophie.jenkins@usz.ch (F.S.J.); esma.yilmaz@med.uni-duesseldorf.de (E.Y.); mohammed.morjan@med.uni-duesseldorf.de (M.M.); amin.thwairan@med.uni-duesseldorf.de (A.T.); johanna.wedy@med.uni-duesseldorf.de (J.W.); georg.holley@stud.semmelweis.hu (G.U.H.); artur.lichtenberg@med.uni-duesseldorf.de (A.L.); hannan.dalyanoglu@med.uni-duesseldorf.de (H.D.); 3 Cardiovascular Research Institute Düsseldorf (CARID), Medical Faculty, Heinrich Heine University, 40225 Düsseldorf, Germany

**Keywords:** MELD, CCI, in-hospital mortality, length of hospitalization, 1-year mortality

## Abstract

Thoracic aortic aneurysms (TAAs) carry a high risk of fatal rupture, necessitating improved preoperative risk stratification. This study evaluates the predictive value of systemic risk scores—specifically the Model for End-Stage Liver Disease (MELD) and the Charlson Comorbidity Index (CCI)—for in-hospital mortality, length of stay, and one-year mortality in patients undergoing elective ascending aortic surgery. The study further compares MELD variants (MELD-Na and MELD-XI) for their prognostic performance in this context. This retrospective single-center study analyzed digital medical records of 500 patients undergoing elective surgery for ascending thoracic aortic disease between 2003 and 2023. MELD, MELD-Na (incorporating sodium), and MELD-XI (excluding INR for anticoagulated patients) were calculated from preoperative laboratory data. The CCI was derived from documented comorbidities. Outcomes included in-hospital mortality, length of stay (from admission to discharge), and one-year mortality assessed via outpatient follow-up. The study excluded patients undergoing emergency surgery for Stanford type A aortic dissection. MELD-Na incorporates serum sodium, while MELD-XI is a variant that excludes INR for patients with anticoagulation. The Charlson Comorbidity Index (CCI) was derived from patients’ medical histories prior to surgery. Length of stay was defined as total inpatient days between admission and discharge. One-year mortality was assessed via outpatient follow-up data. Loss to follow-up did not exceed 30%. Of 500 patients (median age 64 years, 72.8% male), the MELD-Na score showed the strongest ability to predict in-hospital mortality (AUC = 0.698), outperforming both the standard MELD (AUC = 0.690) and the age-adjusted CCI (AUC = 0.631). For one-year mortality (N = 355), MELD-Na again performed best (AUC = 0.732), while the unadjusted CCI showed minimal predictive value (AUC = 0.509). Predictive power for hospital length of stay was limited across all scores; the age-adjusted CCI achieved the highest, though modest, discrimination (AUC = 0.627). 1-year mortality was assessed in 355 patients with available follow-up data (29.0% lost to follow-up). Among these, non-survivors had significantly higher MELD scores (*p* < 0.001). MELD-Na demonstrated the strongest predictive performance (AUC = 0.732). The MELD score, particularly MELD-Na, demonstrated strong predictive ability for in-hospital and 1-year mortality, but showed limited value in estimating hospital stay duration. MELD-Na and the age-adjusted CCI provide valuable preoperative prognostic information for patients undergoing elective ascending aortic surgery. While not intended to replace established risk models, their simplicity and reliance on routine clinical data make them attractive tools for early triage, especially in older or multimorbid patients. Their integration into preoperative planning may enhance individualized risk assessment and resource allocation.

## 1. Introduction

Thoracic aortic aneurysms (TAAs) pose a life-threatening risk due to their potential for rupture or dissection, which are associated with mortality rates exceeding 80% [1,2,3]. Surgical repair, particularly of the ascending aorta and root, remains the definitive treatment, especially when concomitant aortic valve pathology requires composite graft replacement [4].

### 1.1. Timing of Surgical Intervention

In asymptomatic patients, elective surgery is typically recommended when the aortic diameter surpasses defined thresholds, taking into account connective tissue disorders and familial predispositions [4,5]. This decision is increasingly nuanced, as it must also integrate patient-specific risk factors beyond anatomical criteria [3].

### 1.2. Importance of Preoperative Risk Assessment

As the cardiac surgical population becomes older and more multimorbid—due to rising prevalence of hypertension, diabetes, and renal insufficiency—underscoring the growing importance of individualized preoperative risk assessment [5,6]. Particularly in thoracic aortic surgery, where perioperative mortality remains high, systemic patient factors play a critical role in postoperative outcomes [6,7,8]. Existing cardiac risk models such as the EuroSCORE II and the STS Score are widely used but have shown limitations in thoracic aortic pathologies due to their focus on coronary and valvular procedures [9,10,11,12]. Notably, the STS Score does not account for aorta-specific features such as diameter, growth rate, or connective tissue disorders, and was therefore not applied in this study. While the Society of Thoracic Surgeons (STS) Risk Score is an established and widely used tool for perioperative risk assessment in cardiac surgery, its applicability to elective thoracic aortic aneurysm repair is limited. Therefore, despite its general clinical utility, the STS Score was not used in this analysis, as it does not reflect the unique pathophysiological and procedural characteristics of ascending aortic aneurysm surgery. Instead, our study focused on systemic predictors—specifically MELD and age-adjusted CCI—which are designed to capture extracardiac organ dysfunction and overall comorbidity burden, factors increasingly recognized as critical determinants of outcome in complex aortic procedures.

### 1.3. Alternative Risk Stratification Approaches

Originally developed to predict short-term mortality in chronic liver disease, the MELD score reflects systemic dysfunction through parameters such as renal function (creatinine), hepatic function (bilirubin), and coagulation status (INR) [13,14,15,16,17]. Variants such as MELD-Na (with serum sodium) and MELD-XI (without INR) offer improved prognostic value, particularly in patients on anticoagulation. Emerging evidence supports their utility in cardiac surgery, where end-organ dysfunction—especially cardiohepatic interactions—may influence outcomes [18,19]. The Charlson Comorbidity Index (CCI), encompassing 19 comorbidity categories, has demonstrated prognostic relevance in cardiovascular surgery [20,21,22]. However, its predictive accuracy in thoracic aortic surgery remains insufficiently characterized.

### 1.4. Study Objective

This study evaluates the prognostic utility of MELD and CCI scores—including MELD-Na and MELD-XI—for predicting in-hospital mortality, length of hospitalization, and one-year mortality in patients undergoing elective ascending aortic surgery. We hypothesize that these systemic scores may provide complementary risk information beyond classical cardiac models.

If validated, these scores could support early outpatient triage and preoperative decision-making, especially in elderly or multimorbid patients where classical risk models fall short.

## 2. Materials and Methods

### 2.1. Study Population

This retrospective single-center study included 500 consecutive patients who underwent elective surgery for ascending aortic pathologies at the University Hospital Düsseldorf between January 2003 and December 2023. Patients undergoing emergency surgery for Stanford type A dissection, redo operations, or with incomplete laboratory data required for score calculation (creatinine, bilirubin, INR, sodium) were excluded. The long recruitment period reflects the low incidence of elective ascending aortic surgery and the need to generate a sufficiently powered cohort.

### 2.2. Study Design and Data Sources

MELD, MELD-Na (which includes serum sodium), and MELD-XI (which excludes INR for anticoagulated patients) were calculated using preoperative laboratory data. Patients with missing values for any score component were excluded from the respective subanalyses. The Charlson Comorbidity Index (CCI) was calculated both as unadjusted and age-adjusted versions based on documented comorbidities prior to surgery. The unadjusted CCI was analyzed as a categorical variable to reflect clinical risk strata, while the age-adjusted CCI was treated as a continuous variable to preserve statistical granularity.

Primary endpoints were in-hospital mortality, total length of stay (LOS, in days), and one-year mortality. Length of stay was defined as the number of inpatient days from admission to discharge. For subgroup analysis, patients were stratified into tertiles by LOS (short, intermediate, long), based on the distribution in the study population. Stroke was assessed as a secondary outcome but was not the focus of this risk model evaluation. One-year mortality was assessed via structured outpatient follow-up, medical records, and telephone contact where necessary.

### 2.3. Ethics

The study was approved by the local ethics committee (Heinrich Heine University Düsseldorf, Protocol #2023-2566, 6 December 2023) and conducted in accordance with the Declaration of Helsinki. Due to the retrospective and anonymized design, patient consent was waived.

### 2.4. Statistical Analysis

MELD scores were calculated from a single preoperative laboratory measurement, reflecting clinical practice in elective surgery planning. Missing data were handled via listwise deletion for the respective scores. Interactions between MELD and CCI were explored but did not reach statistical significance. Categorical variables were compared using Pearson’s χ^2^ test or Fisher’s exact test, as appropriate for expected frequencies. Non-normally distributed continuous variables were compared via Kruskal-Wallis and Mann-Whitney U tests. Normality of continuous variables was assessed using the Shapiro-Wilk test and visual inspection of histograms and Q-Q plots.

Although scatter plots suggested non-linear patterns, Pearson correlation was used to assess linear associations in line with prior literature. As a sensitivity analysis, Spearman’s rank correlation coefficients (ρ) were additionally calculated to assess potential monotonic, non-linear relationships. Both correlation methods yielded consistent results. The limitations of this approach are acknowledged in the discussion. Survival probabilities were evaluated using Kaplan-Meier methodology with log-rank testing for between-group differences. Receiver operating characteristic (ROC) curve analyses were used to assess the discriminative ability of each risk score for the defined endpoints. Differences between AUCs were compared using the DeLong test for correlated ROC curves. All pairwise comparisons were conducted between MELD, MELD-Na, MELD-XI, and age-adjusted CCI. Non-significant differences were reported for transparency. Optimal cut-off values for sensitivity and specificity were determined using the Youden index. One-year mortality was primarily analyzed as a binary outcome at a fixed time point (365 days); therefore, standard ROC analysis was applied. In addition, to account for time dependency of the outcome during the one-year mortality period, discriminatory power of the risk scores was also assessed with Harrell’s C-Index. Significant associations remained after adjustment. Given the observational design and predefined score variables, no propensity-matching was performed.

All analyses were performed using R statistical software (v4.2.1; R Foundation), with two-tailed *p* < 0.05 considered statistically significant.

## 3. Results

### 3.1. Patient Characteristics

Of the 500 patients included, 72.8% were male, with a median age of 64 years (IQR: 54–73). The most frequent comorbidity was arterial hypertension (82.8%), followed by a history of smoking (36%). The median EuroSCORE II was 3.9, and the median MELD score was 7.3. Baseline characteristics are summarized in Table 1.

### 3.2. MELD Score

The median MELD score was 7.3 (IQR: 5.4, 10.0). The distribution of MELD scores was as follows: 372 (74.4%) patients had a MELD score < 10, 89 (17.8%) patients had a MELD score between 10 and 19, and 39 (7.8%) patients had a MELD score > 19. The median MELD score excluding INR (MELD-XI) was 7.3 (IQR: 5.3, 9.8), and the median MELD-Na score including sodium was also 7.3 (IQR: 5.4, 10.5) (Table 1).

### 3.3. Charlson Comorbidity Index

Using the Charlson Comorbidity Index (CCI) without age adjustment, the most frequent score was 2, observed in 264 patients (52.8%), followed by a score of 3 in 87 patients (17.4%). The remaining distribution was as follows: 1 (82 patients, 16.4%), 4 (43 patients, 8.6%), and 5 (18 patients, 3.6%). Less than 1.2% of patients had a CCI score ≥ 6. The median age-adjusted CCI was 4 (IQR: 3, 5) (Table 1). The age-adjusted CCI yielded a median of 4, indicating a moderate-to-high-comorbidity burden in this surgical population (Table 1).

### 3.4. In-Hospital Mortality

In-hospital mortality was 7.8% (39/500). Non-survivors were significantly older (median age 70 vs. 63 years; *p* = 0.001), had higher MELD scores (median 11.9 vs. 6.9; *p* < 0.001), and a greater prevalence of total arch replacement (*p* = 0.001). MELD-Na and MELD-XI also differed significantly between groups. While the unadjusted CCI showed no significant association with mortality (*p* = 0.4), the Age-adjusted CCI was higher in non-survivors (*p* = 0.006). The Age-adjusted CCI was also significantly higher in non-survivors compared to survivors (5 vs. 4, *p* = 0.006), while the unadjusted CCI did not distinguish survivors from non-survivors (*p* = 0.400). The EuroSCORE II was significantly higher in non-survivors (median 12%) compared to survivors (median 3.7%; *p* ≤ 0.001) (Table 2).

### 3.5. Receiver Operating Characteristics Curves In-Hospital Mortality

**Figure 1:**
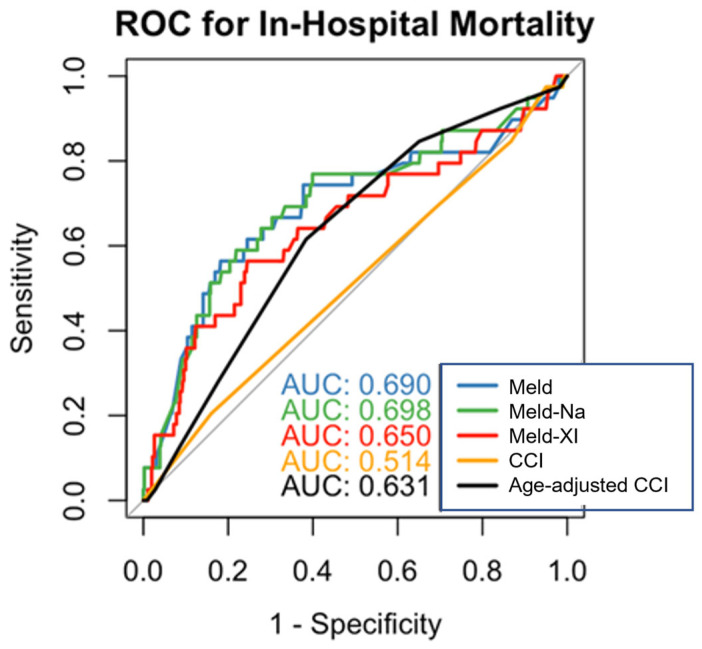
ROC curves for in-hospital mortality prediction (MELD, MELD-Na, MELD-XI, CCI, age-adjusted CCI).

ROC curve analysis demonstrated that the MELD score had a high discriminatory ability for predicting in-hospital mortality, with an AUC of 0.690. Compared to MELD-Na (AUC = 0.698) and MELD-XI (AUC = 0.690), the age-adjusted CCI performed moderately (AUC = 0.631). The unadjusted CCI was not predictive (AUC = 0.514), while the age-adjusted CCI had a higher AUC (AUC = 0.631) (Figure 1). Pairwise comparison of AUCs using the DeLong test revealed no statistically significant differences between MELD, MELD-Na, and MELD-XI (all *p* > 0.05). EuroSCORE II was not plotted due to missing values in 9.4% of cases.

### 3.6. Time of Hospitalization

The median length of stay was 12 days. MELD and MELD-Na scores were slightly higher in patients with prolonged stays (*p* = 0.005 and *p* = 0.006, respectively) (Table 3), while MELD-XI did not show significant differences. The age-adjusted CCI was significantly associated with prolonged hospitalization (*p* < 0.001), suggesting that age-related comorbidity burden may influence postoperative recovery time. The EuroSCORE II was significantly higher in the longest-stay group (median: 5.0) compared to the shortest-stay group (median: 3.1; *p* ≤ 0.001).

### 3.7. Receiver Operating Characteristic (ROC) Curves for Length of Hospital Stay

**Figure 2:**
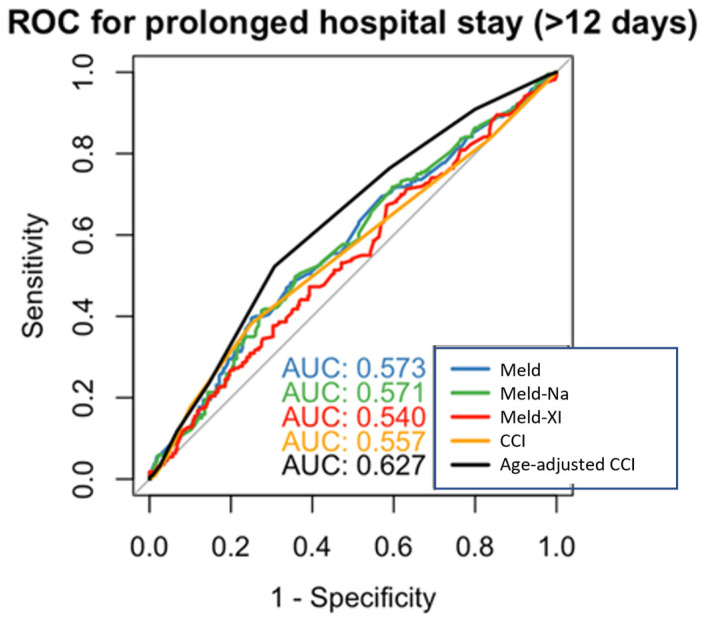
ROC curves for prediction of prolonged hospital stay (MELD, MELD-Na, MELD-XI, CCI, age-adjusted CCI).

ROC curve analysis demonstrated that the MELD score showed an acceptable but not high discriminatory ability for predicting length of hospital stay (AUC = 0.573), as did MELD-Na (AUC = 0.571). MELD-XI (AUC = 0.540). and the unadjusted CCI showed a low discriminatory ability (AUC = 0.557), while the age-adjusted CCI performed better (AUC = 0.627) (Figure 2). DeLong testing for AUC comparison showed no statistically significant differences among MELD, MELD-Na, MELD-XI, and age-adjusted CCI (all *p* > 0.05).

**Figure 3:**
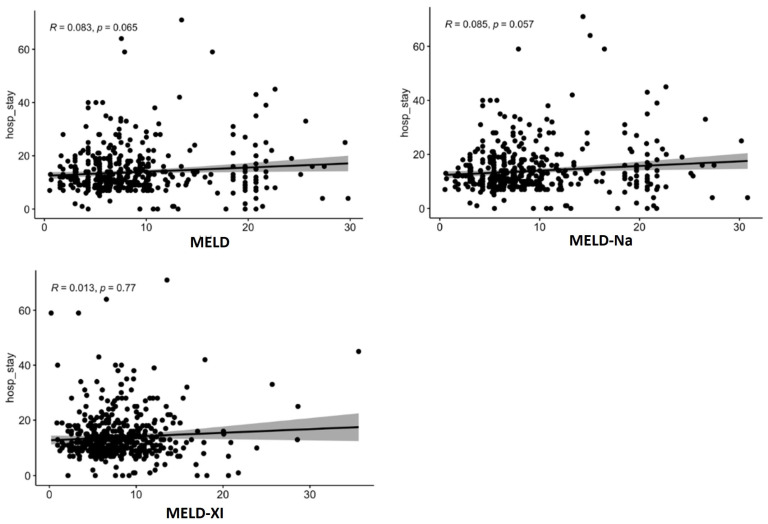
Scatter plots for MELD for length of hospital stay (includes in-hospital deaths).

The scatter plots (Figure 3) depict the relationship between the Model for End-Stage Liver Disease (MELD) scores (MELD, MELD-Na, and MELD_XI) and the length of hospital stay (hosp_stay). Each plot shows a weak positive correlation between the MELD score and the length of stay, with correlation coefficients (R) of 0.083, 0.085, and 0.013, respectively. The *p*-values associated with these correlations are 0.065, 0.057, and 0.77, none of which are statistically significant at the conventional 0.05 level. This suggests that there is no strong statistical evidence of a linear relationship between MELD scores and hospital stay duration based on these plots. The data points are widely dispersed, indicating substantial variability in hospital stay lengths across all MELD score ranges (Figure 3). In sensitivity analyses using Spearman’s rank correlation, results were consistent with the Pearson coefficients: ρ = 0.080 for MELD (*p* = 0.07), ρ = 0.083 for MELD-Na (*p* = 0.06), and ρ = 0.010 for MELD-XI (*p* = 0.78). None reached statistical significance.

**Figure 4:**
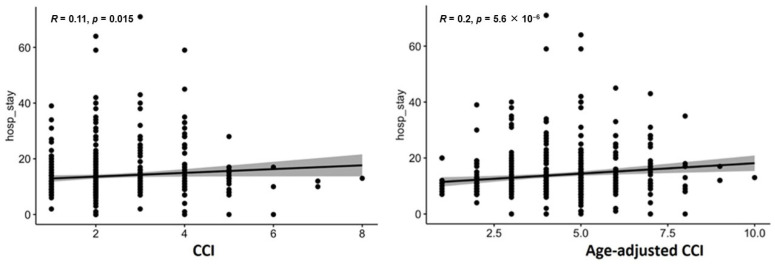
Scatter plots for CCI for length of hospital stay (includes in-hospital deaths).

The scatter plots (Figure 4) explore the relationship between the Charlson Comorbidity Index (CCI) and Age-adjusted CCI, and the length of hospital stay (hosp_stay), including in-hospital deaths. Both plots suggest a weak positive correlation. The correlation coefficient (R) for hosp_stay and CCI is 0.11 with a *p*-value of 0.015, while the correlation between hosp_stay and Age-adjusted CCI is 0.2, with a *p*-value of 5.6 × 10^−6^. The association between hosp_stay and Age-adjusted CCI is more statistically significant than that of hosp_stay and CCI (Figure 4). Spearman’s correlations confirmed these findings (ρ = 0.10, *p* = 0.02 for CCI; ρ = 0.19, *p* < 0.001 for age-adjusted CCI).

**Figure 5:**
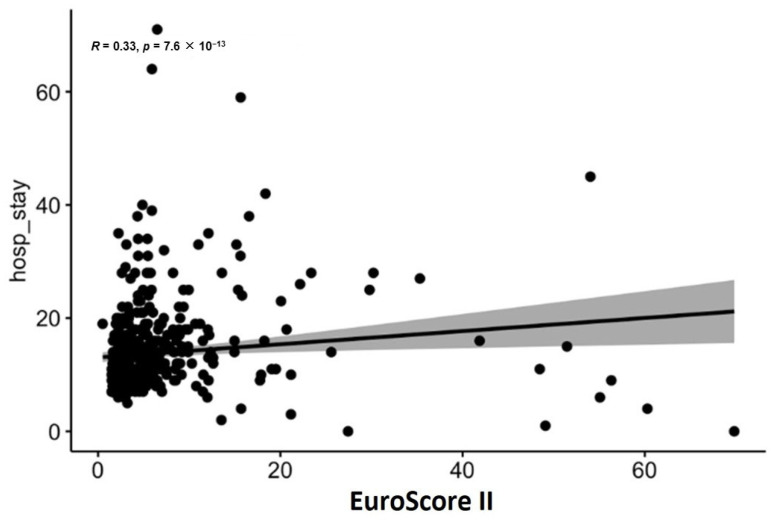
Scatter plot for EuroSCORE II for length of hospital stay (includes in-hospital deaths).

The scatter plot (Figure 5) shows a weak positive correlation between EuroSCORE II and the length of hospital stay, with a correlation coefficient of 0.33 and a highly significant *p*-value of 7.6 × 10^−13^. This suggests that higher EuroSCORE II values are slightly associated with longer hospital stays.

Scatter plots confirmed weak correlations between MELD scores and length of stay (R < 0.1, *p* > 0.05). Age-adjusted CCI showed a stronger association (R = 0.2, *p* < 0.001), while EuroSCORE II correlated modestly (R = 0.33, *p* < 0.001). The Spearman correlation for EuroSCORE II and length of hospital stay was ρ = 0.32 (*p* < 0.001), indicating a similar magnitude of association as the Pearson coefficient. A detailed comparison of Pearson and Spearman correlation coefficients for all analyzed risk scores is presented in Table 4.

### 3.8. 1-Year Mortality

Of the 500 patients, follow-up data were available for 355 (71.0%), while 145 (29.0%) were lost to follow-up. This subgroup formed the basis for the 1-year mortality analysis (9.2%). Non-survivors were significantly older than survivors (*p* ≤ 0.001) (Table 5).

MELD score was significantly higher in non-survivors (*p* ≤ 0.001), and similar results were observed for MELD-Na (*p* ≤ 0.001) and MELD-XI (*p* ≤ 0.001). The CCI did not differ significantly between groups (*p* = 0.2), while the Age-adjusted CCI was higher in non-survivors (*p* ≤ 0.001). The EuroSCORE II was significantly higher in non-survivors (*p* ≤ 0.001). Patients lost to follow-up did not differ significantly in baseline characteristics.

### 3.9. Receiver Operating Characteristics Curves 1-Year Mortality

**Figure 6:**
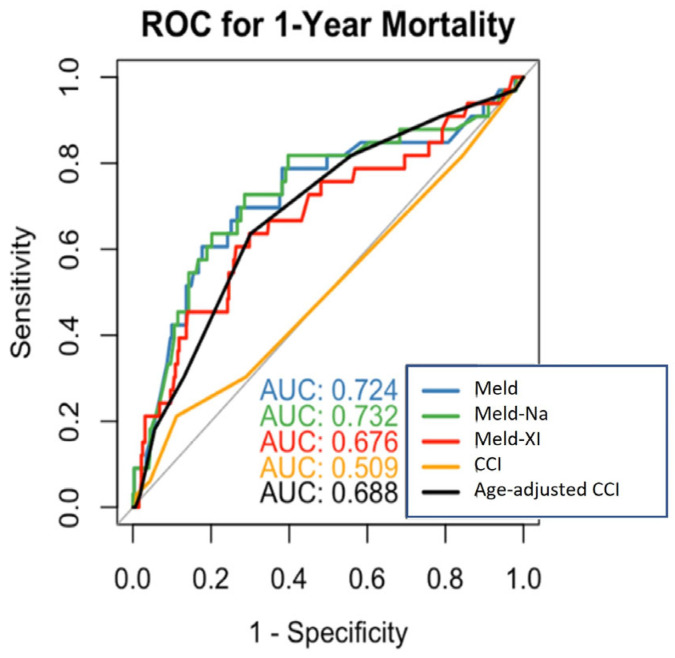
ROC curves for 1-year mortality prediction (MELD, MELD-Na, MELD-XI, CCI and Age-adjusted CCI).

ROC curve analysis demonstrated a high discriminatory ability of the MELD score for predicting 1-year mortality (AUC = 0.724). MELD-Na (AUC = 0.732) and MELD-XI (AUC = 0.676) showed similarly high discriminatory abilities. The CCI showed insufficient discriminatory ability (AUC = 0.509) (Figure 6). Pairwise comparison of AUCs using the DeLong test confirmed that differences between MELD, MELD-Na, and MELD-XI were not statistically significant (*p* > 0.05). Time-dependent analysis of one-year mortality yielded similar discriminatory values for the MELD and CCI scores, with a C-index of 0.701 for MELD Na, 0.688 for MELD, 0.615 for MELD-XI, 0.512 for the CCI without age, and 0.657 for the CCI with age.

### 3.10. Survival Time Analysis

**Figure 7:**
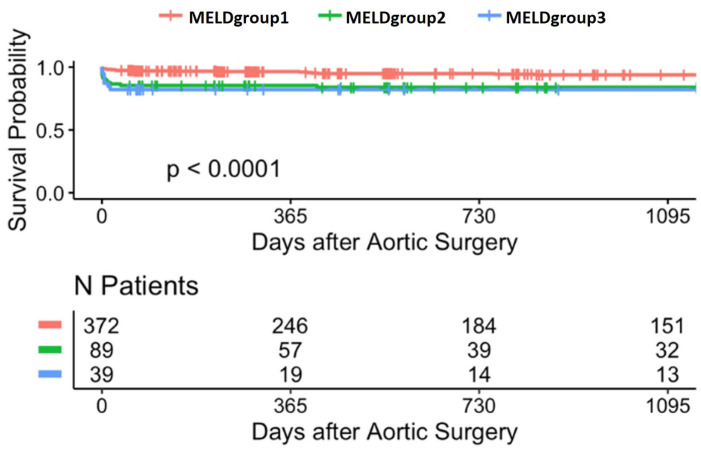
Kaplan-Meier survival curves stratified by MELD group (3-year follow-up).

The Kaplan-Meier survival analysis for MELD groups reveals a statistically significant difference in survival probability (*p* < 0.0001) following aortic surgery. Patients were stratified into three groups based on their MELD score. The number of patients at risk decreased over time, with distinct survival probabilities among groups over a 1095-day follow-up period (Figure 7).

In order to further characterize the survivor cohort according to MELD risk stratification, patients were divided into three groups based on their MELDgroups 1–3 scores (<10, 10–19, >19). Table 6 provides a detailed comparison of demographic, clinical, and risk score parameters among these MELDgroups 1–3. Notably, patients in the highest MELDgroup3 (>19) tended to be older and exhibited a higher prevalence of comorbidities, as well as elevated risk scores, compared to those in the lower MELDgroup1 and 2 categories.

**Figure 8:**
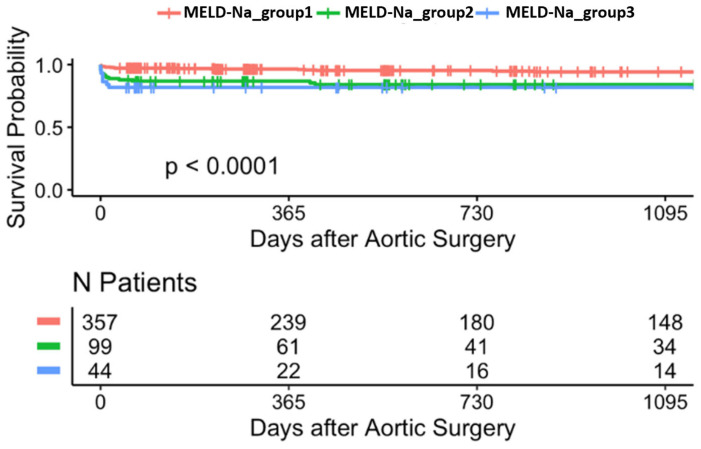
Kaplan-Meier survival curves stratified by MELD-Na_groups (3-year follow-up).

Analysis of the MELD-Na groups using Kaplan–Meier curves (Figure 8) demonstrates a significant association with survival probability post-aortic surgery (*p* < 0.0001). The stratification into three MELD-Na groups highlights differing survival outcomes, with a clear decrease in the number of patients at risk in each group as the follow-up period extends to 1095 days.

**Figure 9:**
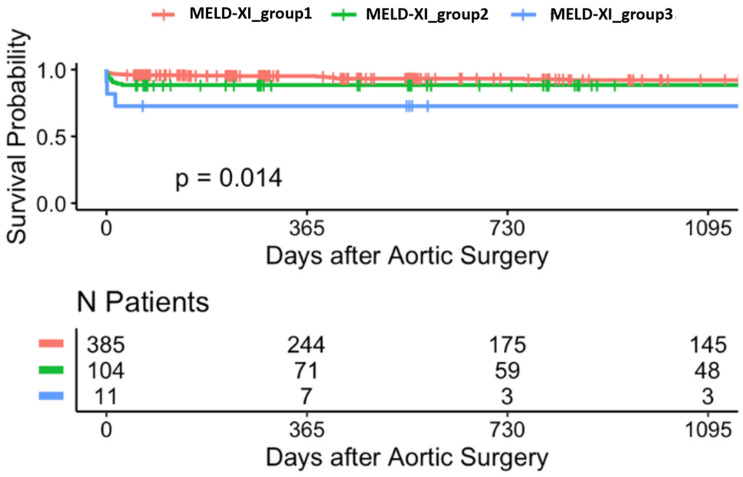
Kaplan-Meier survival curves stratified by MELD XI group (3-year follow-up).

The Kaplan-Meier survival curves for the MELD-XI groups (Figure 9) indicate a statistically significant difference in survival probabilities (*p* = 0.014) after aortic surgery. The number of patients at risk in each of the three MELD-XI based groups changes over the 1095-day study duration, reflecting different survival rates among the groups.

Log-rank testing showed significant survival differences across all MELD groups (*p* < 0.0001). Cox regression models were not included due to violation of proportional hazards assumption in sensitivity testing.

## 4. Discussion

As outlined in the introduction, this study evaluated the prognostic value of MELD and CCI scores in elective ascending aortic surgery, aiming to complement existing cardiac risk models.

In particular, we evaluated the performance of the MELD score and the Charlson Comorbidity Index (CCI) for preoperative risk stratification in a high-risk, elective surgical population.

Surgical intervention, particularly replacement of the ascending aorta using a composite graft prosthesis, remains the primary treatment strategy. The decision to operate on asymptomatic TAAs requires careful weighing of surgical risks against the likelihood of rupture, considering aortic diameter, connective tissue disorders, and individual patient characteristics. An increasing number of elderly cardiac surgery patients with multiple comorbidities highlights the urgent need for improved risk assessment in thoracic aortic surgery, where complication rates are notably high. While the EuroSCORE remains a fundamental tool for assessing cardiac risk, it shows limitations in the context of thoracic aortic diseases. The Model for End-Stage Liver Disease (MELD) score, initially developed to predict survival in liver disease, reflects systemic dysfunction and has shown promise in cardiac surgery, including aortic dissection. The Charlson Comorbidity Index (CCI) is useful in cardiovascular risk assessment, but its effectiveness in thoracic aortic surgery requires further investigation. This study aims to evaluate the MELD score and the CCI as predictors of outcomes in patients undergoing elective surgery for ascending aortic aneurysms, with the goal of improving preoperative decision-making in this high-risk patient population.

### 4.1. Prognostic Significance of the MELD Score and Charlson Comorbidity Index (CCI)

This study provides novel insights into the prognostic utility of the MELD score and the Charlson Comorbidity Index (CCI) in a high-risk cohort undergoing thoracic aortic surgery. Originally developed to prioritize liver transplant candidates based on short-term mortality risk [23,24], the predictive value of the MELD score is increasingly recognized in non-hepatic settings, including cardiac surgery. Our findings confirm this broader applicability, showing that higher MELD scores are associated with increased in-hospital and one-year mortality, and to a lesser extent, with prolonged hospital length of stay.

### 4.2. MELD and Mortality in Cardiac Surgery: Expanding the Evidence

The MELD score was significantly higher among patients who died during hospitalization (median: 11.9 vs. 6.9, *p* < 0.001), and showed moderate to high discriminative performance for in-hospital mortality (AUC = 0.690) and one-year mortality (AUC = 0.724). These findings align with recent studies indicating that MELD scores ≥13 are predictive of early postoperative mortality in various cardiac surgeries, including valve and coronary artery bypass operations [25,26]. This supports the notion that hepatic congestion and right heart dysfunction—common in this cohort—may drive perioperative risk more than previously acknowledged. The superior performance of MELD-Na (AUC = 0.732) over standard MELD (AUC = 0.724) supports the hypothesis that hyponatremia—often a marker of chronic volume overload and right heart dysfunction—adds significant prognostic value. However, when formally compared using the DeLong test, these AUC differences did not reach statistical significance (*p* > 0.05), indicating that MELD-Na, MELD, and MELD-XI perform comparably in this population. Kim et al. [27] demonstrated that MELD-Na improves mortality prediction in cirrhotic patients, and our results suggest that this improvement extends to cardiac populations, particularly those with hepatic congestion due to chronic right heart failure or tricuspid regurgitation [28,29].

The prognostic value of MELD-XI is particularly relevant in anticoagulated patients, which constitute a significant subset in cardiac surgery. Its robustness across INR variability enhances its clinical utility in real-world settings (AUC = 0.676 for one-year mortality). This is particularly relevant in cardiac surgery patients, many of whom are on vitamin K antagonists or direct oral anticoagulants, which may distort INR-based liver function assessments. Previous studies have found similar results and support the utility of MELD-XI in cardiac and transplant settings where INR may be pharmacologically influenced [18,19]. Given the widespread use of anticoagulants in cardiac patients, MELD-XI may serve as a more stable alternative when INR is not reliable.

### 4.3. MELD and Hospital Length of Stay: Limited Predictive Power

As expected, MELD and CCI scores demonstrated only limited utility in predicting hospital length of stay (AUC: MELD 0.573, MELD-Na 0.571, MELD-XI 0.540). This reflects the multifactorial nature of prolonged hospitalization, which may be more strongly influenced by surgical complexity, perioperative complications, and rehabilitation capacity than by preoperative organ function. Scatter plots showed only weak, statistically non-significant correlations between MELD scores and hospital stay duration (R = 0.083–0.085, *p* > 0.05), suggesting that factors beyond hepatic and renal function—such as surgical complexity, postoperative complications, and rehabilitation needs—play a more decisive role in determining length of stay. These findings differ from liver transplantation populations, in which MELD often correlates more strongly with hospital resource utilization [27]. Sensitivity analyses using Spearman’s rank correlation confirmed the robustness of these results, yielding comparable coefficients and identical levels of statistical significance.

### 4.4. Charlson Comorbidity Index: Importance of Age Adjustment

The Charlson Comorbidity Index (CCI), although widely validated for predicting long-term mortality in various clinical settings [30], performed poorly in its unadjusted form (AUC = 0.514 for in-hospital mortality). However, the age-adjusted CCI significantly outperformed the unadjusted version (AUC = 0.631), underscoring the central role of age in evaluating surgical risk. Non-survivors were significantly older than survivors in both in-hospital and one-year mortality analyses (*p* < 0.001), confirming previous findings that operative risk is amplified not only by comorbidity burden but also by age-related vulnerability [31,32]. The age-adjusted CCI also showed a significant association with prolonged hospital stay and one-year mortality, supporting its value as a complementary tool in risk assessment among older patients undergoing complex cardiac surgery. Our data confirm that age is a key driver of postoperative risk in thoracic aortic surgery and should be integrated into any predictive framework. The poor performance of the unadjusted CCI supports prior findings and validates the use of its age-adjusted version [31,32].

### 4.5. Comparison with Established Risk Models: MELD vs. EuroSCORE II

EuroSCORE II, an established cardiac risk model, was more strongly associated with in-hospital and one-year mortality than MELD or CCI (*p* < 0.001 for all comparisons). However, EuroSCORE II requires detailed perioperative variables—such as left ventricular function, operative urgency, and hemodynamic status—which may not be readily available during enabling early outpatient risk stratification during preoperative planning or in semi-urgent scenarios. In contrast, MELD-Na and age-adjusted CCI rely solely on readily available laboratory and clinical information, allowing for early outpatient risk stratification—even in time-critical or resource-limited settings [9,33].

### 4.6. Existing Risk Stratification Models for Ascending Aortic Surgery

While MELD and its derivatives show predictive power, their utility must be interpreted within the broader framework of risk stratification in thoracic aortic surgery. Traditional models such as EuroSCORE II, although validated across a wide spectrum of cardiac surgeries, may not fully capture the unique hemodynamic and systemic complexities inherent to aortic procedures [34]. Risk models specifically developed for ascending aortic surgery include the GERAADA Score, derived from the German Registry for Acute Aortic Dissection Type A, which incorporates parameters such as malperfusion, consciousness, and cardiac tamponade. This model demonstrates good discriminatory performance (AUC~0.80) but is limited to acute dissection and lacks validation in elective settings [35]. Similarly, the IRAD Risk Score uses data from the International Registry of Acute Aortic Dissection, stratifying risk via clinical variables like hypotension, pulse deficits, and renal dysfunction. While informative in emergency dissection, it is less applicable in chronic or elective aortic interventions [36].

The Cleveland Clinic models provide robust assessments for thoracic aortic surgery, incorporating surgical details, renal function, and ventricular performance, achieving AUC values exceeding 0.75. However, like EuroSCORE II, their complexity and need for detailed data can hinder practical application in urgent scenarios [37].

The Society of Thoracic Surgeons (STS) Risk Score, while widely used for estimating perioperative risk in cardiac surgery, was not specifically designed for aortic procedures. It primarily incorporates variables relevant to coronary artery bypass grafting and valvular surgery, such as left ventricular function, prior cardiac interventions, renal function, and pulmonary comorbidities. As previously discussed, the STS Score lacks aortic-specific parameters and is therefore limited in its applicability to elective thoracic aortic surgery. Most established aortic risk scores, such as GERAADA and IRAD, were developed for acute type A dissections and do not apply to elective scenarios. Similarly, the STS Score—while validated for coronary and valvular surgery—lacks aortic-specific parameters. Therefore, MELD and CCI may serve as valuable adjuncts to existing models by capturing extracardiac organ dysfunction.

### 4.7. The Role of Systemic Organ Dysfunction in Aortic Surgery

What differentiates MELD and CCI from classical cardiac models is their ability to quantify systemic dysfunction, particularly involving hepatic and renal systems. In our cohort, MELD-Na outperformed standard MELD and MELD-XI in predicting both short- and long-term mortality, likely due to the added prognostic value of hyponatremia—a marker of chronic heart failure and hepatic congestion. This systemic dysfunction is often underrepresented in traditional cardiac risk tools. Moreover, the weak performance of the unadjusted CCI emphasizes the importance of age-adjusted risk profiling in older populations, where age interacts with comorbidity to substantially elevate perioperative risk. MELD captures aspects of cardiohepatic syndrome, a condition increasingly recognized in advanced cardiovascular disease [36,37]. Elevated central venous pressure may impair hepatic function, reflected in abnormal bilirubin and INR levels. Serial measurements of MELD—pre- and postoperative—may enhance prognostication, as suggested by recent findings in ECMO patients [24].

### 4.8. Toward Integrated Risk Models

Our findings, alongside recent literature, support the development of integrated risk models that combine established cardiac predictors (e.g., EuroSCORE II, GERAADA) with systemic indices such as MELD-Na and age-adjusted CCI. These findings argue for a paradigm shift toward integrated, systems-based risk models that combine established cardiovascular indices (e.g., EuroSCORE II) with markers of hepatic and renal dysfunction. Such hybrid approaches may better reflect the multisystemic nature of risk in major aortic surgery. For example, a multicenter analysis from 2021 demonstrated that combining EuroSCORE II with markers of hepatic and renal dysfunction significantly improved 30-day mortality prediction following aortic root replacement [38]. Likewise, Tse et al. (2022) showed that inclusion of liver function parameters enhanced the accuracy of aortic surgery mortality prediction, particularly in patients with right heart dysfunction or congestive hepatopathy [39]. A practical example would be a triage algorithm incorporating EuroSCORE II and MELD-Na, especially for older patients with hepatic congestion. Such approaches reflect the growing understanding that thoracic aortic surgery outcomes are not only determined by cardiac anatomy and surgical technique, but also by the functional reserves of other vital organs.

### 4.9. Pathophysiological Considerations: The Cardiohepatic Axis

A potential explanation for MELD’s predictive value lies in the cardiohepatic axis. Chronic right heart failure and elevated central venous pressure cause hepatic congestion, fibrosis, and impaired synthetic function—reflected in elevated bilirubin and INR, both central MELD components [40,41]. This mechanism highlights how cardiac dysfunction may manifest in hepatic laboratory derangements, reinforcing the utility of MELD and MELD-Na in this population.

Renal dysfunction and sodium imbalance further elevate MELD scores, often due to fluid overload, diuretic use, or systemic inflammation, conditions commonly seen in patients with advanced aortic pathology. These inter-organ dynamics argue for broader adoption of systemic scoring tools in high-risk surgical patients. Serial MELD measurements, particularly preoperatively and on postoperative day 1, could capture dynamic risk changes and should be explored in future studies.

### 4.10. Clinical Implementation and Decision-Making Integration

The integration of MELD-Na and age-adjusted CCI into clinical workflows offers a pragmatic approach to early risk stratification in elective thoracic aortic surgery. Both scores rely on routinely available data-basic laboratory values and documented comorbidities-making them suitable for use in outpatient settings or during initial surgical evaluation. In practice, these scores could be incorporated into preoperative checklists or electronic health record (EHR)-based decision support tools to flag high-risk patients early in the planning process.

For example, patients with MELD-Na ≥ 13 or age-adjusted CCI ≥ 5 could be automatically referred for multidisciplinary case review, including anesthesiology, geriatrics, and intensive care specialists. This would allow for tailored perioperative planning, such as prehabilitation, optimization of volume status, or early ICU bed reservation. Furthermore, these scores could complement existing models like EuroSCORE II by providing a systemic risk perspective, particularly in patients with preserved cardiac function but significant extracardiac vulnerability [42].

### 4.11. Health Economic Implications and Resource Allocation

From a health economics perspective, the use of MELD-Na and age-adjusted CCI may support more efficient allocation of perioperative resources, especially in aging and multimorbid populations. Identifying patients at elevated risk for prolonged hospitalization or postoperative complications enables targeted deployment of high-cost interventions such as intensive monitoring, early mobilization programs, or specialized nursing care.

Moreover, in systems with constrained ICU or step-down unit capacity, these scores could inform triage decisions and help prioritize surgical scheduling. For instance, lower-risk patients (MELD-Na < 10, CCI < 3) may be suitable for fast-track recovery protocols, while higher-risk individuals may benefit from enhanced recovery pathways or even reconsideration of surgical indication in borderline cases. This stratified approach could reduce avoidable complications, shorten length of stay, and ultimately lower overall treatment costs without compromising patient safety [43].

### 4.12. Limitations and Future Directions

This study has several limitations that must be acknowledged. First, the retrospective, single-center design limits the generalizability of our findings and may introduce selection bias. Patients with incomplete laboratory data were excluded from score calculation, which may have skewed the risk distribution. Moreover, score components were obtained from a single preoperative timepoint, potentially overlooking dynamic organ function changes that occur perioperatively. Due to the limited number of events and the exploratory nature of this study, multivariable modeling was not performed. Future studies should explore automated variable selection methods (e.g., AIC, LASSO) and assess model performance using Harrell’s C-index and proportional hazards testing.

Second, the relatively high loss to follow-up at one year (29%) may have affected the accuracy of long-term mortality estimates. Although baseline characteristics did not differ significantly between patients with and without follow-up, an attrition bias cannot be excluded. Additionally, the long study period (2003–2023) raises concerns about temporal variability in surgical techniques, perioperative management, and laboratory measurement standards.

Third, the MELD and CCI scores were not originally developed for cardiac surgery populations, and validated thresholds for elective thoracic aortic procedures are lacking. The clinical applicability of these scores, particularly in terms of decision-making algorithms or triage pathways, remains to be defined. Also, interactions between MELD, EuroSCORE II, and other comorbidity indices were not fully explored.

Future research should have the following aims:

- Validate the prognostic utility of MELD-Na and age-adjusted CCI in **multicenter, prospective** cohorts of patients undergoing elective thoracic aortic surgery;
- Define **clinically meaningful cut-off values** for MELD-based scores in this setting;
- Investigate the value of **serial MELD measurements** in the perioperative course;
- Explore the development of **hybrid risk models** integrating MELD, EuroSCORE II, and aorta-specific anatomical parameters;
- Assess score performance across different surgical techniques (e.g., hemiarch vs. full arch replacement).

## 5. Conclusions

In this study, the MELD-Na score and age-adjusted Charlson Comorbidity Index (CCI) demonstrated significant prognostic utility for predicting in-hospital and one-year mortality in patients undergoing elective ascending aortic surgery. Both scores are derived from routinely available clinical and laboratory data, making them simple and accessible tools for early-stage preoperative risk assessment.

While these indices are not designed to replace established cardiac risk models such as EuroSCORE II or GERAADA, they provide complementary systemic perspectives by capturing hepatic, renal, and age-related vulnerability-factors increasingly recognized as critical in major aortic procedures.

Integrating these scores into multimodal risk stratification frameworks may improve individualized surgical planning, particularly in older and comorbid patients. Future guidelines should consider incorporating MELD-Na and age-adjusted CCI to support patient-centered decision-making in elective aortic surgery.

## Figures and Tables

**Table 1:** Baseline Characteristics, Comorbidities, and Preoperative Risk Scores of the Study Cohort N = 500.

Characteristic	N = 500 ^1^
Sex	
Men	364 (72.8%)
Women	136 (27.2%)
BMI ^2^	26.3 (23.9, 29.2)
Age	64 (54, 73)
CPR ever ^3^	10 (2.0%)
MI ever ^4^	28 (5.6%)
Smoking ever ^5^	182 (36.4%)
Diabetes	51 (10.2%)
Hyperlipidemia	168 (33.6%)
AHTN ^6^	414 (82.8%)
ASA classification ^7^	
1	20 (4.0%)
2	21 (4.2%)
3	392 (78.4%)
4	64 (12.8%)
5	3 (0.6%)
Aorta diameter	50 (46, 57) ^8^
Previous surgery ^9^	54 (10.8%)
Endocarditis active	28 (5.6%)
Marfan syndrome	4 (0.8%)
Preoperative troponin ^10^	15 (8, 27) ^10^
Surgery duration ^11^	240 (191, 304) ^11^
In-hospital death	39 (7.8%)
Hospital stay ^12^	12 (9, 16) ^12^
Euroscore II	3.9 (2.5, 6.3)
CCI simple ^13^	
1	82 (16.4%)
2	264 (52.8%)
3	87 (17.4%)
4	43 (8.6%)
5	18 (3.6%)
6	3 (0.6%)
7	2 (0.4%)
8	1 (0.2%)
CCI age ^14^	4.00 (3.00, 5.00)
MELD-I ^15^	7.3 (5.4, 10.0)
MELD-II ^16^	7.3 (5.4, 10.5)
MELD-XI ^17^	7.3 (5.3, 9.8)
MELD-I group ^18^	
1	372 (74.4%)
2	89 (17.8%)
3	39 (7.8%)
MELD-II group ^19^	
1	357 (71.4%)
2	99 (19.8%)
3	44 (8.8%)
MELD-XI group ^20^	
1	385 (77.0%)
2	104 (20.8%)
3	11 (2.2%)
1-year mortality	
0	322 (64.4%)
1	33 (6.6%)
Lost to follow-up	145 (29.0%)

^1^ n (%); Median (Q1, Q3), ^2^ body mass index, ^3^ cardiopulmonary resuscitation in past medical history, ^4^ myocardial infarction in past medical history, ^5^ smoking active or in past medical history, ^6^ arterial hypertension, ^7^ ASA physical status classification system, ^8^ in millimeters, ^9^ previous cardiac surgery, ^10^ in nanograms per liter, ^11^ in minutes, ^12^ in days, ^13^ unadjusted Charlson Comorbidity Index (CCI) in absolute CCI points, ^14^ age-adjusted CCI in absolute CCI points, ^15^ Model for End-Stage Liver Disease (MELD), ^16^ MELD including sodium levels (MELD-Na), ^17^ MELD excluding INR, ^18^ MELD I, ^19^ MELD-Na, and ^20^ MELD-XI were each grouped into three risk categories: scores < 10 = group 1, scores 10–19 = group 2, and scores > 19 = group 3.

**Table 2:** Comparison of Demographic, Clinical, and Risk Score Parameters Between In-Hospital Survivors and Non-Survivors.

Variable	Overall N = 500 ^1^	Survivors N = 461 ^1^	Non-Survivors N = 39 ^1^	*p*-Value ^2^
Sex				0.2
Men	364 (72.8%)	332 (72.0%)	32 (82.0%)	
Women	136 (27.2%)	129 (27.9%)	7 (17.5%)	
BMI ^3^	26.3 (23.9, 29.2)	26.3 (23.9, 29.2)	26.3 (24.5, 30.4)	0.7
Age ^4^	64 (54, 73) ^4^	63 (53, 72) ^4^	70 (65, 76) ^4^	0.001
CPR ever ^5^	10 (2.0%)	4 (0.9%)	6 (15%)	<0.001
MI ever ^6^	28 (5.6%)	27 (5.9%)	1 (2.6%)	0.7
Smoking ever ^7^	182 (36.4%)	172 (37.3%)	10 (25.6%)	0.15
Diabetes	51 (10.2%)	44 (9.5%)	7 (17.9%)	0.10
Hyperlipidemia	168 (33.6 4%)	150 (32.5%)	18 (46.2%)	0.084
AHTN ^8^	414 (82.8%)	387 (83.9%)	27 (69.2%)	0.019
ASA classification ^9^				0.006
1	20 (4.0%)	17 (3.7%)	3 (7.7%)	
2	21 (4.2%)	19 (4.1%)	2 (5.1%)	
3	392 (78.4%)	368 (79.8%)	24 (61.5%)	
4	64 (12.8%)	56 (12.1%)	8 (20.5%)	
5	3 (0.6%)	1 (0.2%)	2 (5.1%)	
Aorta diameter ^10^	50 (46, 57) ^10^	51 (46, 57) ^10^	47 (38, 50) ^10^	0.007
Unknown diameter	97	80	17	
Previous surgery ^11^	54 (10.8%)	46 (9.9%)	8 (20.5%)	0.056
Endocarditis active	28 (5.6%)	18 (3.9%)	10 (25.6%)	<0.001
Marfan syndrome	4 (0.8%)	4 (0.9%)	0 (0.0%)	>0.900
Preop. troponin ^12^	15 (8, 27) ^12^	14 (8, 25) ^12^	29 (16, 83) ^12^	<0.001
Surgery duration ^13^	240 (191, 304) ^13^	237 (190, 293) ^13^	347 (225, 417) ^13^	<0.001
Hospital stay ^14^	12 (9, 16) ^14^	12 (9, 16) ^14^	9 (1, 14) ^14^	<0.001
Euroscore II	3.9 (2.5, 6.3)	3.7 (2.3, 5.9)	12.0 (5.5, 21.2)	<0.001
Unknown Euroscore II	47	37	10	
CCI ^15^				0.400
1	82 (16.4%)	74 (16.1%)	8 (20.5%)	
2	264 (52.8%)	245 (53.1%)	19 (48.7%)	
3	87 (17.4%)	81 (17.6%)	6 (15.4%)	
4	43 (8.6%)	38 (8.2%)	5 (12.8%)	
5	18 (3.6%)	18 (3.9%)	0 (0.0%)	
6	3 (0.6%)	2 (0.4%)	1 (2.6%)	
7	2 (0.4%)	2 (0.4%)	0 (0.0%)	
8	1 (0.2%)	1 (0.2%)	0 (0.0%)	
Age-adjusted CCI ^16^	4.00 (3.00, 5.00)	4.00 (3.00, 5.00)	5.00 (4.00, 6.00)	0.006
MELD-I ^17^	7.3 (5.4, 10.0)	6.9 (5.4, 9.4)	11.9 (7.0, 19.7)	<0.001
MELD-II ^18^	7.3 (5.4, 10.5)	7.3 (5.4, 10.0)	12.6 (7.5, 19.7)	<0.001
MELD-XI ^19^	7.3 (5.3, 9.8)	7.2 (5.3, 9.5)	9.8 (6.5, 12.7)	0.002
MELD-I group ^20^				<0.001
1	372 (74.4%)	355 (77.0%)	17 (43.6%)	
2	89 (17.8%)	75 (16.3%)	14 (35.9%)	
3	39 (7.8%)	31 (6.7%)	8 (20.5%)	
MELD-II group ^21^				<0.001
1	357 (71.4%)	341 (73.9%)	16 (41.0%)	
2	99 (19.8%)	85 (18.4%)	14 (35.9%)	
3	44 (8.8%)	35 (7.6%)	9 (23.1%)	
MELD-XI group ^22^				0.005
1	385 (77.0%)	363 (78.7%)	22 (56.4%)	
2	104 (20.8%)	89 (19.3%)	15 (38.4%)	
3	11 (2.2%)	9 (2.0%)	2 (5.1%)	
1-year mortality				<0.001
0	322 (64.4%)	313 (67.9%)	9 (23.1%)	
1	33 (6.6%)	3 (0.7%)	30 (76.9%)	
Lost to follow-up	145 (29.0%)	145 (31.4%)	0 (0.0%)	

^1^ n (%); Median (Q1, Q3), ^2^ Pearson’s Chi-squared test; Wilcoxon rank sum test; Fisher’s exact test, ^3^ body mass index, ^4^ in years, ^5^ cardiopulmonary resuscitation in past medical history, ^6^ myocardial infarction in past medical history, ^7^ smoking active or in past medical history, ^8^ arterial hypertension, ^9^ ASA physical status classification system, ^10^ in millimeters, ^11^ previous cardiac surgery, ^12^ in nanograms per liter, ^13^ in minutes, ^14^ in days, ^15^ unadjusted Charlson Comorbidity Index (CCI) in absolute points, ^16^ age-adjusted CCI, in absolute CCI points, ^17^ Model for End-Stage Liver Disease (MELD), ^18^ MELD including sodium levels (MELD-Na), ^19^ MELD excluding INR, ^20^ MELD I, ^21^ MELD-Na, and ^22^ MELD-XI were each grouped into three risk categories: scores < 10 = group 1, scores 10–19 = group 2, and scores > 19 = group 3.

**Table 3:** Comparison of Demographic, Clinical, and Risk Score Parameters by Length of Hospital Stay Group.

Variable	Overall N = 500 ^1^	<12 Days N = 280 ^1^	>12 Days N = 220 ^1^	*p*-Value ^2^
Sex				0.040
Men	364 (72.8%)	214 (7.4%)	150 (68.2%)	
Women	136 (27.2%)	66 (23.6%)	70 (31.8%)	
BMI ^3^	26.3 (23.9, 29.2)	26.1 (23.8, 29.0)	26.9 (24.0, 29.7)	0.3
Age ^4^	64 (54, 73) ^4^	62 (52, 71) ^4^	68 (58, 75) ^4^	<0.001
CPR ever ^5^	10 (2.0%)	7 (2.5%)	3 (1.4%)	0.5
MI ever ^6^	28 (5.6%)	15 (5.4%)	13 (5.9%)	0.8
Smoking ever ^7^	182 (36.4%)	106 (37.8%)	76 (34.5%)	0.4
Diabetes	51 (10.2%)	25 (8.9%)	26 (11.8%)	0.3
Hyperlipidemia	168 (33.6%)	89 (31.8%)	79 (35.9%)	0.3
AHTN ^8^	414 (82.8%)	225 (80.4%)	189 (85.9%)	0.10
ASA classification ^9^				0.011
1	20 (4.0%)	11 (3.9%)	9 (4.1%)	
2	21 (4.2%)	12 (4.3%)	9 (4.1%)	
3	392 (78.4%)	232 (82.8%)	160 (72.7%)	
4	64 (12.8%)	23 (8.2%)	41 (18.6%)	
5	3 (0.6%)	2 (0.7%)	1 (0.5%)	
Aorta diameter ^10^	50 (46, 57) ^10^	50 (46, 55) ^10^	52 (46, 59) ^10^	0.018
Unknown diameter	97	52	45	
Previous surgery ^11^	54 (10.8%)	27 (9.6%)	27 (12.3%)	0.3
Endocarditis active	28 (5.6%)	15 (5.4%)	13 (5.9%)	0.8
Marfan syndrome	4 (0.8%)	4 (1.4%)	0 (0.0%)	0.13
Preop. troponin ^12^	15 (8, 27) ^12^	12 (8, 25) ^12^	19 (10, 34) ^12^	<0.001
Surgery duration ^13^	240 (191, 304) ^13^	240 (189, 292) ^13^	246 (194, 316) ^13^	0.059
In-hospital death	39 (7.8%)	28 (10%)	11 (5.0%)	0.039
Hospital stay ^14^	12 (9, 16) ^14^	9 (8, 11) ^14^	17 (14, 22) ^14^	<0.001
Euroscore II	3.9 (2.5, 6.3)	3.1 (2.2, 4.9)	5.0 (3.1, 8.2)	<0.001
Unknown Euroscore II	47	23	24	
CCI ^15^				
1	82 (16.4%)	46 (16.4%)	36 (16.4%)	
2	264 (52.8%)	164 (58.6%)	100 (45.4%)	
3	87 (17.4%)	42 (15.0%)	45 (20.4%)	
4	43 (8.6%)	16 (5.7%)	27 (12.3%)	
5	18 (3.6%)	8 (2.9%)	10 (4.5%)	
6	3 (0.6%)	2 (0.7%)	1 (0.5%)	
7	2 (0.4%)	2 (0.7%)	0 (0.0%)	
8	1 (0.2%)	0 (0.0%)	1 (0.5%)	
Age-adjusted CCI ^16^	4.00 (3.00, 5.00)	4.00 (3.00, 5.00)	5.00 (4.00, 5.00)	<0.001
MELD ^17^	7.3 (5.4, 10.0)	6.5 (5.3, 9.0)	7.5 (5.4, 10.7)	0.005
MELD-Na ^18^	7.3 (5.4, 10.5)	7.1 (5.4, 9.7)	7.7 (5.8, 11.7)	0.006
MELD-XI ^19^	7.3 (5.3, 9.8)	7.2 (5.2, 9.5)	7.6 (5.4, 10.1)	0.12
MELDgroup ^20^				0.030
1	372 (74.4%)	221 (78.9%)	151 (68.6%)	
2	89 (17.8%)	42 (15.0%)	47 (21.4%)	
3	39 (7.8%)	17 (6.1%)	22 (10.0%)	
MELD-Na_group ^21^				0.019
1	357 (71.4%)	214 (76.4%)	143 (65.0%)	
2	99 (19.8%)	45 (16.1%)	54 (24.5%)	
3	44 (8.8%)	21 (7.5%)	23 (10.4%)	
MELD-XI group ^22^				0.2
1	385 (77.0%)	224 (80.0%)	161 (73.2%)	
2	104 (20.8%)	51 (18.2%)	53 (24.1%)	
3	11 (2.2%)	5 (1.8%)	6 (2.7%)	
1-year mortality				0.002
0	322 (64.4%)	186 (66.4%)	136 (61.8%)	
1	33 (6.6%)	26 (9.3%)	7 (3.2%)	
Lost to follow-up	145 (29.0%)	68 (24.3%)	77 (35.0%)	

^1^ n (%); Median (Q1, Q3), ^2^ Pearson’s Chi-squared test; Wilcoxon rank sum test; Fisher’s exact test, ^3^ body mass index, ^4^ in years, ^5^ cardiopulmonary resuscitation in past medical history, ^6^ myocardial infarction in past medical history, ^7^ smoking active or in past medical history, ^8^ arterial hypertension, ^9^ ASA physical status classification system, ^10^ in millimeters, ^11^ previous cardiac surgery, ^12^ in nanograms per liter, ^13^ in minutes, ^14^ in days, ^15^ unadjusted Charlson Comorbidity Index (CCI) in absolute points, ^16^ Age-adjusted CCI, in absolute CCI points, ^17^ Model for End-Stage Liver Disease (MELD), ^18^ MELD including sodium levels (MELD-Na), ^19^ MELD excluding INR, ^20^ MELD I, ^21^ MELD-Na, and ^22^ MELD-XI were each grouped into three risk categories: scores < 10 = group 1, scores 10–19 = group 2, and scores > 19 = group 3.

**Table 4:** Sensitivity analysis: Spearman’s rank correlation coefficients (ρ) compared to Pearson correlation coefficients (r) for associations between preoperative risk scores and length of hospital stay.

Variable (vs. Length of Stay)	Pearson r	*p*-Value	Spearman ρ	*p*-Value	Interpretation
MELD	0.083	0.07	0.080	0.07	No significant correlation
MELD-Na	0.085	0.06	0.083	0.06	No significant correlation
MELD-XI	0.013	0.78	0.010	0.78	No significant correlation
CCI	0.11	0.02	0.10	0.02	Weak positive correlation
Age-adjusted CCI	0.20	<0.001	0.19	<0.001	Moderate positive correlation
EuroSCORE II	0.33	<0.001	0.32	<0.001	Moderate positive correlation

**Table 5:** Demographic, Clinical, and Risk Score Characteristics by One-Year Mortality Status (n = 355, Patients With Complete Follow-up).

Variable	Overall N = 355 ^1^	Survivors N = 322 ^1^	Non-Survivors N = 33 ^1^	*p*-Value ^2^
Sex				0.8
Men	273 (76.9%)	247 (76.7 7%)	26 (78.8%)	
Women	82 (23.1%)	75 (23.3%)	7 (21.2%)	
BMI ^3^	26.2 (23.7, 29.0)	26.1 (23.7, 29.0)	26.2 (24.1, 28.7)	0.8
Age ^4^	61 (51, 70) ^4^	59 (51, 69) ^4^	70 (67, 75) ^4^	<0.001
CPR ever ^5^	9 (2.5%)	3 (0.9%)	6 (18.2%)	<0.001
MI ever ^6^	14 (3.9%)	13 (4.0%)	1 (3.0%)	>0.9
Smoking ever ^7^	139 (39.2%)	130 (40.4%)	9 (27.3%)	0.14
Diabetes	27 (7.6%)	22 (6.8%)	5 (15.2%)	0.092
Hyperlipidemia	118 (33.2%)	106 (32.9%)	12 (36.4%)	0.7
AHTN ^8^	284 (80.0%)	263 (81.7%)	21 (63.6%)	0.014
ASA classification ^9^				<0.001
1	16 (4.5%)	13 (4.0%)	3 (9.1%)	
2	15 (4.2%)	14 (4.3%)	1 (3.0%)	
3	278 (78.3%)	260 (80.7%)	18 (54.5%)	
4	43 (12.1%)	34 (10.5%)	9 (27.3%)	
5	3 (0.8%)	1 (0.3%)	2 (6.1%)	
Aorta diameter ^10^	50 (46, 56) ^10^	50 (46, 56) ^10^	47 (38, 57) ^10^	0.11
Unknown diameter	73	58	15	
Previous surgery ^11^	40 (11.3%)	32 (9.9%)	8 (24.2%)	0.021
Endocarditis active	23 (6.5%)	13 (4.0%)	10 (30.3%)	<0.001
Marfan syndrome	4 (1.1%)	4 (1.2%)	0 (0.0%)	>0.9
Preoperative troponin ^12^	15 (8, 29) ^12^	14 (8, 26) ^12^	39 (16, 84) ^12^	<0.001
Surgery duration ^13^	239 (190, 304) ^13^	231 (187, 288) ^13^	365 (266, 503) ^13^	<0.001
In-hospital death	39 (10.9%)	9 (2.8%)	30 (90.9%)	<0.001
Hospital stay ^14^	11 (9, 16) ^13^	12 (9, 16) ^13^	4 (1, 12) ^13^	<0.001
Euroscore II	3.4 (2.2, 6.0)	3.3 (2.2, 5.4)	15.4 (5.9, 41.9)	<0.001
Unknown Euroscore II	35	23	12	
CCI ^15^				0.2
1	56 (15.7%)	50 (15.5 6%)	6 (18.2%)	
2	196 (55.2%)	179 (55.6%)	17 (51.5%)	
3	60 (16.9%)	57 (17.7%)	3 (9.1%)	
4	27 (7.6%)	22 (6.8%)	5 (15.2%)	
5	12 (3.4%)	11 (3.4%)	1 (3.0%)	
6	2 (0.6%)	1 (0.3%)	1 (3.0%)	
7	1 (0.3%)	1 (0.3%)	0 (0%)	
8	1 (0.3%)	1 (0.3%)	0 (0%)	
Age adjusted CCI ^16^	4.00 (3.00, 5.00)	4.00 (3.00, 5.00)	5.00 (4.00, 6.00)	<0.001
MELD ^17^	7.3 (5.4, 10.2)	7.0 (5.4, 9.7)	12.6 (7.6, 19.7)	<0.001
MELD-Na ^18^	7.3 (5.4, 10.4)	7.3 (5.4, 10.0)	12.7 (8.1, 19.7)	<0.001
MELD-XI ^19^	7.4 (5.4, 10.1)	7.2 (5.4, 9.8)	9.9 (7.3, 12.7)	<0.001
MELD-group ^20^				<0.001
1	259 (72.9%)	246 (76.4%)	13 (39.4%)	
2	70 (19.7 20%)	57 (17.7%)	13 (39.4%)	
3	26 (7.3%)	19 (5.9%)	7 (21.2%)	
MELD-Na group ^21^				<0.001
1	251 (7.7%)	239 (74.2%)	12 (36.4%)	
2	74 (20.8%)	61 (18.9%)	13 (39.4%)	
3	30 (8.5%)	22 (6.8%)	8 (24.2%)	
MELD-XI group ^22^				0.009
1	262 (73.8%)	244 (75.7%)	18 (54.5%)	
2	83 (23.4%)	71 (22.1%)	12 (36.4%)	
3	10 (2.8%)	7 (2.2%)	3 (9.1%)	

^1^ n (%); Median (Q1, Q3), ^2^ Pearson’s Chi-squared test; Wilcoxon rank sum test; Fisher’s exact test, ^3^ body mass index, ^4^ in years, ^5^ cardiopulmonary resuscitation in past medical history, ^6^ myocardial infarction in past medical history, ^7^ smoking active or in past medical history, ^8^ arterial hypertension, ^9^ ASA physical status classification system, ^10^ in millimeters, ^11^ previous cardiac surgery, ^12^ in nanograms per liter, ^13^ in minutes, ^14^ in days, ^15^ unadjusted Charlson Comorbidity Index (CCI) in absolute points, ^16^ age-adjusted CCI, in absolute CCI points, ^17^ Model for End-Stage Liver Disease (MELD), ^18^ MELD including sodium levels (MELD-Na), ^19^ MELD excluding INR, ^20^ MELD I, ^21^ MELD-Na, and ^22^ MELD-XI were each grouped into three risk categories: scores < 10 = group 1, scores 10–19 = group 2, and scores > 19 = group 3.

**Table 6:** Comparison of Demographic, Clinical, and Risk Score Parameters by MELD-I Group (Survivors Only).

Variable	Overall N = 500 ^1^	<10 N = 372 ^1^	10–19 N = 89 ^1^	>19 N = 39 ^1^	*p*-Value ^2^
Sex					0.011
Men	364 (72.8%)	258 (69%)	75 (84%)	31 (79%)	
Women	136 (27.2%)	114 (31%)	14 (16%)	8 (21%)	
BMI ^3^	26.3 (23.9, 29.2)	26.2 (23.7, 29.1)	26.6 (24.2, 29.4)	27.5 (24.7, 29.4)	0.3
Age ^4^	64 (54, 73) ^4^	63 (53, 71) ^4^	68 (56, 74) ^4^	72 (58, 77) ^4^	<0.001
CPR ever ^5^	10 (2.0%)	5 (1.3%)	1 (1.1%)	4 (10%)	0.008
MI ever ^6^	28 (5.6%)	21 (5.6%)	4 (4.5%)	3 (7.7%)	0.7
Smoking ever ^7^	182 (36.4%)	142 (38%)	29 (33%)	11 (28%)	0.3
Diabetes	51 (10.2%)	33 (8.9%)	13 (15%)	5 (13%)	0.2
Hyperlipidemia	168 (33.6%)	128 (34%)	27 (30%)	13 (33%)	0.8
AHTN ^8^	414 (82.8%)	311 (84%)	72 (81%)	31 (79%)	0.7
ASA classification ^9^					
1	20 (4.0%)	14 (3.8%)	6 (6.7%)	0 (0.0%)	
2	21 (4.2%)	20 (5.4%)	1 (1.1%)	0 (0.0%)	
3	392 (78.4%)	302 (81.2%)	63 (70.8%)	27 (69.2%)	
4	64 (12.8%)	34 (9.1%)	18 (20.2%)	12 (30.7%)	
5	3 (0.6%)	2 (0.5%)	1 (1.1%)	0 (0.0%)	
Aorta diameter ^10^	50 (46, 57) ^10^	51 (46, 56) ^10^	50 (46, 55) ^10^	56 (46, 60) ^10^	0.6
Unknown diameter	97	62	18	17	
Previous surgery ^11^	54 (10.8%)	31 (8.3%)	14 (16%)	9 (23.1%)	0.006
Endocarditis active	28 (5.6%)	13 (3.5%)	8 (9.0%)	7 (17.9%)	<0.001
Marfan syndrome	4 (0.8%)	3 (0.8%)	1 (1.1%)	0 (0%)	0.7
Preop. troponin ^12^	15 (8, 27) ^12^	12 (8, 22) ^12^	24 (11, 41) ^12^	41 (21, 68) ^12^	<0.001
Surgery duration ^13^	240 (191, 304) ^13^	234 (189, 290) ^13^	247 (201, 341) ^13^	271 (195, 356) ^13^	0.004
In-hospital death	39 (7.8%)	17 (4.6%)	14 (16%)	8 (21%)	<0.001
Hospital stay ^14^	12 (9, 16) ^14^	11 (9, 16) ^14^	13 (9, 16) ^14^	15 (8, 20) ^14^	0.2
Euroscore II	3.9 (2.5, 6.3)	3.4 (2.2, 5.3)	5.4 (3.0, 12.3)	9.2 (5.9, 17.9)	<0.001
Unknown Euroscore II	47	23	14	10	
**CCI** ^15^					
1	82 (16.4%)	63 (16.9%)	14 (15.7%)	5 (12.8%)	
2	264 (52.8%)	214 (57.5%)	37 (41.6%)	13 (33.3%)	
3	87 (17.4%)	59 (15.9%)	19 (21.3%)	9 (23.1%)	
4	43 (8.6%)	24 (6.5%)	10 (11.2%)	9 (23.1%)	
5	18 (3.6%)	9 (2.4%)	6 (6.7%)	3 (7.7%)	
6	3 (0.6%)	1 (0.3%)	2 (2.2%)	0 (0.0%)	
7	2 (0.4%)	2 (0.5%)	0 (0.0%)	0 (0.0%)	
8	1 (0.2%)	0 (0.0%)	1 (1.1%)	0 (0.0%)	
Age-adjusted CCI ^16^	4.00 (3.00, 5.00)	4.00 (3.00, 5.00)	4.00 (3.00, 6.00)	5.00 (4.00, 6.00)	<0.001
MELD ^17^	7.3 (5.4) 10.0)	6.4 (4.9, 7.4)	12.6 (10.7, 18.5)	20.8 (20.8, 22.6)	<0.001
MELD-Na ^18^	7.3 (5.4) 10.5)	6.4 (5.1, 7.5)	13.1 (10.9, 18.5)	21.4 (20.8, 22.6)	<0.001
MELD-XI ^19^	7.3 (5.3) 9.8)	6.6 (4.9, 8.3)	9.9 (7.3, 13.0)	12.2 (9.4, 17.0)	<0.001
MELD-Na group ^20^					
1	357 (71.4%)	357 (95.9%)	0 (0.0%)	0 (0.0%)	
2	99 (19.8%)	15 (4.0%)	84 (94.4%)	0 (0.0%)	
3	44 (8.8%)	0 (0.0%)	5 (5.6%)	39 (100.0%)	
MELD-XI group ^21^					<0.001
1	385 (77.0%)	327 (87.9%)	46 (51.7%)	12 (30.1%)	
2	104 (20.8%)	45 (12.1%)	39 (43.8%)	20 (51.3%)	
3	11 (2.2%)	0 (0.0%)	4 (4.5%)	7 (17.9%)	
1-year mortality					<0.001
0	322 (64.4%)	246 (66.1%)	57 (64.0%)	19 (8.7%)	
1	33 (6.6%)	13 (3.5%)	13 (14.6%)	7 (17.9%)	
Lost to follow-up	145 (29.0%)	113 (30.4%)	19 (21.3%)	13 (33.3%)	

^1^ n (%); Median (Q1, Q3), ^2^ Pearson’s Chi-squared test; Wilcoxon rank sum test; Fisher’s exact test, ^3^ body mass index, ^4^ in years, ^5^ cardiopulmonary resuscitation in past medical history, ^6^ myocardial infarction in past medical history, ^7^ smoking active or in past medical history, ^8^ arterial hypertension, ^9^ ASA physical status classification system, ^10^ in millimeters, ^11^ previous cardiac surgery, ^12^ in nanograms per liter, ^13^ in minutes, ^14^ in days, ^15^ unadjusted Charlson Comorbidity Index (CCI) in absolute CCI points, ^16^ Age-adjusted CCI, in absolute CCI points, ^17^ Model for End-Stage Liver Disease (MELD), ^18^ MELD including sodium levels (MELD-Na), ^19^ MELD excluding INR, ^20^ MELD-Na, and ^21^ MELD-XI were each grouped into three risk categories: scores < 10 = group 1, scores 10–19 = group 2, and scores > 19 = group 3.

## Data Availability

The data underlying this study were obtained retrospectively from clinical records and contain sensitive personal health information. Due to privacy regulations and ethical considerations, the raw data cannot be made publicly available. Researchers with a legitimate interest may request access to anonymized or aggregated data, subject to approval by the responsible ethics committee and in compliance with applicable data protection laws.
